# Preeclampsia with Intrauterine Growth Restriction Generates Morphological Changes in Endothelial Cells Associated with Mitochondrial Swelling—An In Vitro Study

**DOI:** 10.3390/jcm8111994

**Published:** 2019-11-15

**Authors:** Dorota Formanowicz, Agnieszka Malińska, Marcin Nowicki, Katarzyna Kowalska, Karolina Gruca-Stryjak, Grzegorz Bręborowicz, Katarzyna Korybalska

**Affiliations:** 1 Department of Clinical Biochemistry and Laboratory Medicine, Poznan University of Medical Sciences, 60-806 Poznań, Poland; 2 Department of Histology and Embryology, Poznan University of Medical Sciences, 60-781 Poznań, Poland; 3 Department of Perinatology and Gynecology, Poznan University of Medical Sciences, 60-535 Poznań, Poland; karolagruca@poczta.onet.pl (K.G.-S.); gbrebor@ump.edu.pl (G.B.); 4 Department of Pathophysiology, Poznan University of Medical Sciences, 60-806 Poznań, Poland; koryb@ump.edu.pl

**Keywords:** IUGR, preeclampsia, endothelial cells, mitochondria

## Abstract

Pregnancy complicated by preeclampsia (PE) and intrauterine growth restriction (IUGR) promotes endothelial cell (EC) dysfunction. Our in vitro study aimed to evaluate the endothelial cell morphology after acute and chronic exposition to medium supplemented with serum taken from healthy pregnant women and women with IUGR and IUGR with PE. In the same condition, ECs viability, proliferation, reactive oxygen species (ROS) production, and serum concentration of vascular endothelial growth factor (VEGF) were also measured. Pregnant women with IUGR and IUGR with PE-delivered babies with reduced body mass and were characterized in elevated blood pressure, urine protein loss, and reduced level of VEGF. The 24 hours of exposition did not exert any morphological changes in ECs, except the reduction in cell viability, but prolonged exposition resulted in significant morphological changes concerning mostly the swelling of mitochondria with accompanying ROS production, cell autophagy, reduced cell viability, and proliferation only in complicated pregnancies. In conclusion, the sera taken from women with IUGR and IUGR with PE show a detrimental effect on ECs, reducing their viability, proliferation, and generating oxidative stress due to dysfunctional mitochondria. This multidirectional effect might have an adverse impact on the cardiovascular system in women with IUGR and PE.

## 1. Introduction

Preeclampsia is a hypertensive disease that complicates 2–8% of pregnancies characterized by high blood pressure and signs of damage to another organ system, most often the liver and kidneys. It is postulated that, in preeclampsia, hypoxia/reperfusion, which generates reactive oxygen species (ROS) production and an apoptotic/necrotic cascade causes morphological changes in the syncytium of preeclamptic placentas. Roland et al. describe many morphological changes of placental syncytium and their implications for the pathogenesis of this complex disorder [1,2]. This pathological alteration promotes the increased release of soluble syncytial factors, including cytokines, eicosanoids, peroxides, the antiangiogenic factor-soluble fms-like tyrosine kinase (sFlt)-1, and soluble endoglin, as well as syncytiotrophoblast microparticles. These factors are suggested to promote maternal endothelial cells (ECs) dysfunction and are associated with placental damage in pregnancies also complicated with intrauterine growth restriction (IUGR) [3,4]. Excess soluble fms-like tyrosine kinase 1 (sFlt-1) binds placental growth factor (PIGF) and vascular endothelial growth factor (VEGF), preventing their interaction with receptors located on ECs [5]. Lack of endothelial response to angiogenic stimuli results in an increased risk of future predisposition to placental disease and later development of vascular disease [6]. Soluble endoglin deleteriously affects vascular tone by blocking the activation of endothelial nitric oxide synthase (eNOS), changing the vascular tone [7]. Yinon et al. demonstrated that vascular function differs between women with early-onset of preeclampsia (PE), late-onset of PE, and normotensive intrauterine growth restriction (IUGR). The authors found reduced flow-mediated dilatation (FMD), and increased arterial stiffness in women with early-onset of PE and IUGR [8]. These findings remain in compliance with the fact that ECs dysfunction is characteristic for both IUGR and IUGR together with PE. However, changes in circulating angiogenic and angiostatic factors in women with a complicated pregnancy, also responsible for ECs dysfunction, are not consistent [8,9]. Yinon et al. did not observe any changes of angiogenic and angiostatic factors in any stage of PE and IUGR [8]; on the contrary, Crispi et al. showed significant differences between them [9]. As reported by Roberts et al., the morphology of human umbilical vein endothelial cells (HUVECs) was minimally altered after exposure to sera of a preeclamptic woman. Therefore, authors concluded that tested sera rather activate ECs than are toxic for them [10]. Preeclamptic sera exerted diverse effects on ECs in vitro: (i) increased expression of fibronectin, (ii) had no impact on von Willebrand factor and tissue factor, (iii) decreased eNOS expression, (iv) increased inducible nitric oxide synthase (iNOS) expression, (v) augmented superoxide anion production, (iv) increased ECs permeability that was induced by IL-8 but not by eNOS [10,11,12].

In this present paper, based on extensive medical reports that show ECs dysfunction in IUGR and IUGR complicated with PE, we evaluated the morphological abnormalities in endothelium after being exposed to sera taken from pregnancies complicated with PE and PE with IUGR. The novelty of our data is to show the variety of changes in ECs ultrastructure, visualized by the electron microscope.

## 2. Experimental Section

### 2.1. Ethics Statement

This study was carried out in accordance with the Declaration of Helsinki of the World Medical Association and approved by the Ethical Committee of Poznan University of Medical Sciences (Decision number: 1212/16). All included study participants fulfilled the criteria and completed the study. They were fully informed about the research, and all of them gave written informed consent before their examination.

### 2.2. Study Design

In the years 2017–2018, a total of 215 women in the third trimester of pregnancy, aged 18–48, were admitted to the Department of Perinatology and Gynaecology at the Poznan University of Medical Sciences or were treated in outpatient clinics. All consecutive pregnant women underwent a careful interview and a clinical examination, and their history was evaluated on the basis of available hospital and outpatient data. Each time, specialist gynecologists, using well-known recognized criteria based on symptoms, laboratory results and imaging tests, qualified a woman into one of three research groups, i.e. (i) control group—healthy women with normal fetal weight, (ii) IUGR group—women with estimated fetal weight at or below the 10th percentile for gestational age but without preeclampsia (PE), (iii) IUGR + PE group—women who met the criteria for PE and IUGR.

The exclusion criteria from this study were: no written consent to be included in the study (*n* = 20); deficiencies in medical records (*n* = 3); chronic disease (*n* = 26); multiple pregnancy (*n* = 27); taking medications during pregnancy that may affect the coagulation system, e.g., acetylsalicylic acid, heparin (*n* = 18); intrauterine growth retardation due to structural defects, fetal genetic abnormalities or intrauterine infection (*n* = 18); smoking and/or alcohol abuse in the past 3 years (*n* = 13) and withdrawal of consent to participate in the study (*n* = 6). In addition, haemolysis was revealed in 8 blood samples.

Finally, a total of 74 pregnant women were qualified for this study; 25 healthy women formed the control group, 25 women with IUGR assessed by specialist doctors formed the IUGR group, and 24 pregnant women with PE associated IUGR, were qualified to the group named IUGR + PE.

### 2.3. Biochemical Analyses

To minimize diurnal variations, fasting blood samples were always collected between 7.30 and 9.00 am. All routine biochemical analyses were performed immediately in a central hospital laboratory. Samples of serum were aliquoted and stored at −80 °C until assayed. The VEGF, was measured by using DuoSet Immunoassay Development Kits (R&D Systems, Minneapolis, MN, USA) according to the manufacturer’s instructions. C-reactive protein (CRP) was measured by using a high sensitivity assay from DRG Medtek (Warsow, Poland). The sensitivity of the assays was: 12.2 pg/mL for VEGF, and 0.1 mg/mL for high-sensitivity C-reactive protein (hsCRP).

### 2.4. Cell Culture

The tests were performed using HUVEC line EA.hy926 (kindly provided by Dr. CJ Edgell, University of North Carolina, Chapel Hill, USA) [13]. The cells were routinely maintained in the Earl’s-buffered M199 culture medium, supplemented with amphotericin (2.5 μg/mL), gentamycin (50 μg/mL), L-glutamine (2 mmol/L), hydrocortisone (0.4 μg/mL), and 10% *v/v* fetal calf serum (Invitrogen, USA). The cultures were maintained at 37 ºC in a humidified atmosphere of 95% air and 5% CO_2._

All the reagents were purchased from Sigma-Aldrich (Saint Louis, MO, USA). Cell culture plastics came from Nunc (Roskilde, Denmark).

### 2.5. Experimental Design

HUVECs were exposed to the culture medium supplemented with 10% *v/v* fetal calf serum (FCS) and 10% human pooled serum for one and 10 days. Human pooled serum was composed of an equal volume of serum taken from 22–25 women from the control group (healthy pregnancies, *n* = 25), from pregnant women with IUGR (*n* = 25), and pregnancies with IUGR + PE group (*n* = 24). The created medium was then sterilized using siring filters (0.22 µm pores, Millipore, Burlington, MA, USA). This process protects the endothelial cell culture against potential infection, especially during prolonged exposition (10 days). During a prolonged exposition medium was exchanged every three days. It would have been much better if endothelium had been exposed to each serum per se. However, due to the small amount of serum taken from pregnant women with complicated pregnancies, we decided to prepare one pooled serum to perform all experiments.

During our experiments we used three types of controls. One control consisted of medium supplemented with 10% *v/v* fetal bovine serum, and it was the recommended milieu for HUVEC culture, as described at the beginning of the method section (Con = standard medium). The second control consisted of medium supplemented with 10% *v/v* human serum taken from non-pregnant women with matched age (HS). The third control consisted of medium supplemented with 10% **v/v** human serum taken from healthy pregnant women (HP). Controls second and third are not a physiological milieu for the HUVECs and were made on the purpose of the experiment, which aims to evaluate diverse human serum.

### 2.6. Endothelial Cell Viability

The experiments were performed in 96-well plates. Cells were seeded in following densities depending on the time of the experiment: at 4 × 10^3^ cells/well and 2 × 10^4^ cells/well respectively for 10 days and 24 h of exposition to tested sera. After one and 10 days of exposition to medium supplemented with pooled human sera (Con, IUGR, IUGR + PE), cell viability was measured using the MTT assay, which measures the metabolic conversion of the MTT salt (3-(4,5-dimethylthiazol-2-yl)-2,5-diphenyl-tetrazolinum bromide) by active dehydrogenases derived in this test from endoplasmic reticulum [14]. The test was performed as described previously [15]. Briefly, after one and 10 days of exposition to tested medium, the cells were incubated in a medium containing 1.25 mg/mL of the MTT salt (3-(4,5-dimethylthiazol-2-yl)-2,5-diphenyl-tetrazolium bromide) for 4 h at 37 °C. The formazan product generated was dissolved with the addition of an acidic solution of 20% *w/v* sodium dodecyl sulfate and 50% *v/v* N, N-dimethylformamide. The absorbance of the converted dye was recorded at 595 nm. The data were expressed in two ways, as a percentage of (i) control HS (cells cultured in a medium supplemented with human serum taken from non-pregnant women) and (ii) control HP (cells cultured in a medium supplemented with serum from healthy pregnant women).

### 2.7. Cell Proliferation

After one and 10 days of exposition to human sera, HUVECs proliferation was assessed by [^3^H]-thymidine incorporation. Briefly, cells in 48 well plates were plated at a density of 2 × 10^4^ cells/well_,_ allowed to attach for 24 hours, and then pulsed with [^3^H]-thymidine (1 μCi/mL; Institute of Radioisotopes, Prague, Czech Republic) for 24 hours. After the incubation, the cells were harvested, precipitated with 10% (*w/v*) trichloroacetic acid, and dissolved in 0.1 M NaOH. The radioactivity released was measured in a beta liquid scintillation counter (Wallac Perkin Elmer). The data were expressed in two ways, as a percentage of (i) control HS (cells cultured in a medium supplemented with human serum taken from non-pregnant women) and (ii) control HP (cells cultured in a medium supplemented with serum from healthy pregnant women).

### 2.8. Detection of Reactive Oxygen Species (ROS)

The experiments were performed in 96-well plates. Cells were seeded in following densities depending on the time of the experiment: at 4 × 10^3^ cells/well and 2 × 10^4^ cells/well respectively for 10 days and 24 h of exposition to tested sera. Detection of ROS was assayed by 2’,7’-dichlorodihydrofluorescein diacetate (H2DCFDA) labeling. Briefly, cells (2 × 10^4^) were incubated with 10 μM H2DCFDA (Molecular Probes, Eugene, OR, USA) for 45 min at 37 ºC and then solubilized with the lysis buffer (Promega, Durham, NC, USA). The measure of ROS generation in cells was the degree of fluorescence of the H2DCFDA dye, which under intracellular ROS was oxidized to the fluorescent product Fluorescence emitted by cell lysate was measured in a spectrofluorometer (Thermo Fisher Scientific, Waltham, MA, USA), using wavelengths of 485 and 535 nm for excitation and emission, respectively. The positive control during the experiment consisted of 100 µM H_2_O_2_. The data were expressed in two ways, as a percentage of (i) control HS (cells cultured in a medium supplemented with human serum taken from non-pregnant women) and (ii) control HP (cells cultured in a medium supplemented with serum from healthy pregnant women).

### 2.9. Light Microscopy

ECs were seeded in 24-well plates at 4 × 10^4^ cells/well and incubated with standard medium (Con), and medium supplemented with pooled human sera (HS, HP, IUGR, IUGR + PE) for 10 days. The microphotographs were taken every 1–3 days using the Axio Observer D1 inverted microscope (Zeiss, Oberkochen, Germany) up to the 10th day of exposition.

### 2.10. Electron Microscopy

The experiments were performed in 6-well plates. Cells were seeded at 4 × 10^5^ cells/well and 10^5^ cells/well respectively for one and 10 days of exposition to tested sera. After 1 and 10 days of exposition to standard medium (Con), and medium supplemented with pooled human sera (HS, HP, IUGR, IUGR + PE), ECs were harvested using trypsin/EDTA, centrifuged, and subsequently fixed in 0.1 M phosphate-buffered saline (PBS) containing 2.5% glutaraldehyde for 1 hour at room temperature, post-fixed in 1% osmium tetraoxide for 30 minutes at room temperature, washed and dehydrated in graded series of ethanol solutions (40–100%). Then, cells were infiltrated and embedded in Epon (Plano, Marburg, Germany). Ultrathin sections (40–60 nm) were mounted on mesh copper grids, contrasted with 2% aqueous uranyl acetate (30 minutes), and lead citrate (30 minutes) and analyzed with a Jeol transmission electron microscope at 80 kV (Jeol, Tokio, Japan).

### 2.11. Statistical Analysis

Statistical analysis was performed using GraphPad PrismTM 6.00 (GraphPad Software Inc, La Jolla, CA, USA). The data were interpreted with repeated measures analysis of variance—one-way ANOVA—using a post hoc test for multiple comparisons (Dunn’s or Tukey’s tests). A *p*-value < 0.05 was considered significant. The data were expressed as means ± standard deviation (SD) or median (min–max).

## 3. Results

### 3.1. Patient Description

Women with complicated pregnancies delivered babies with reduced newborn weight when compared to women without complications. The IUGR and IUGR + PE group was also characterized by higher systolic and diastolic blood pressure and higher urine protein loss. We detected about four times higher levels of VEGF in women with healthy non-complicated pregnancies in contrast to IUGR and IUGR with PE groups. For the characteristics of the study participants see Table 1. Although patients were slightly different in gestational age, no statistically significant differences were found. In addition, we have also checked whether in any analyzes the gestational age had an impact on the obtained results (correlations). No statistically significant relationships were obtained. The results of these analyses showed that the primary selection of patients into the studied groups could be supported, since the groups, especially IUGR and IUGR + PE, were not numerous.

### 3.2. Endothelial Cell Viability and Proliferation

**Figure 1:**
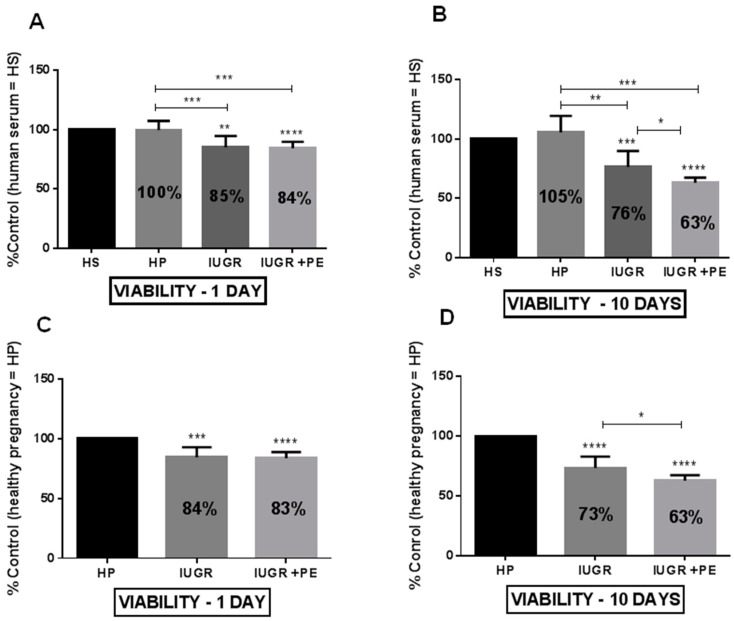
Endothelial cell viability. (**A**) Endothelial cell viability after one day of exposition to medium supplemented with tested sera, expressed as a percent of control which was the human serum taken from non-pregnant women with matched age(HS); (**B**) Endothelial cell viability after ten days of exposition to medium supplemented with tested sera, expressed as a percent of control HS (**C**) Endothelial cell viability after one day of exposition to medium supplemented with tested sera, expressed as a percent of control which was the human serum taken from healthy pregnant women (HP); (**D**) Endothelial cell viability after ten days of exposition to medium supplemented with tested sera, expressed as a percent of control HP. Abbreviations: IUGR—pregnant women with intrauterine growth restriction; IUGR + PE—pregnant woman with both intrauterine growth restriction and preeclampsia. The data were interpreted with repeated measures analysis of variance (one way ANOVA) using a post hoc test for multiple comparisons (Dunn’s or Tukey’s tests). The results are expressed as mean ± standard deviation (SD), derived from 2 independent experiments, and were calculated as a percent of control HS (panel A and B) and control HP (panel C and D). Asterisks represent a significant difference when compared with the representative control cells: *p < 0.05, ** *p* < 0.01, *** *p* < 0.001, **** *p* < 0.0001.

**Figure 2:**
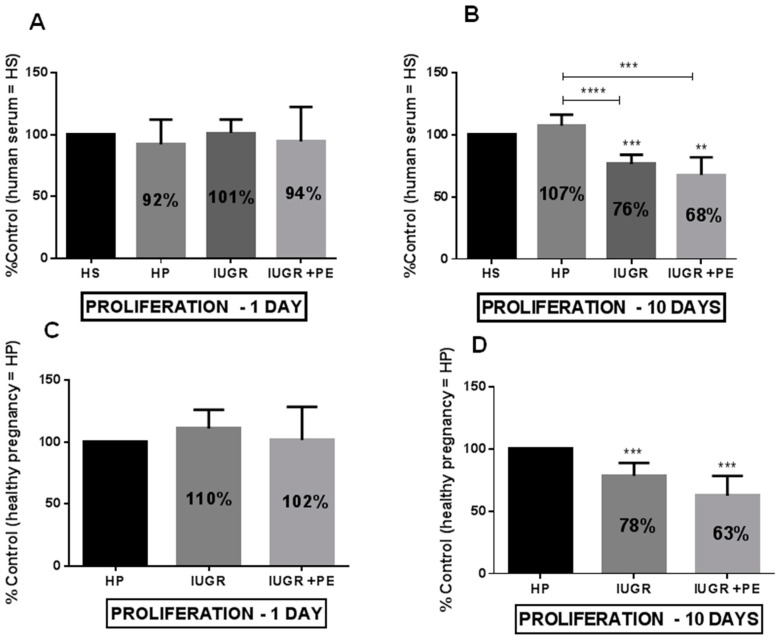
Endothelial cell proliferation (**A**) Endothelial cell proliferation after one day of exposition to medium supplemented with tested sera, expressed as a percent of control which was the human serum taken from non-pregnant women with matched age(HS); (**B**) Endothelial cell proliferation after ten days of exposition to medium supplemented with tested sera, expressed as a percent of control HS (**C**) Endothelial cell proliferation after one day of exposition to medium supplemented with tested sera, expressed as a percent of control which was the human serum taken from healthy pregnant women (HP); (**D**) Endothelial cell proliferation after ten days of exposition to medium supplemented with tested sera, expressed as a percent of control HP. Abbreviations: IUGR—pregnant women with intrauterine growth restriction; IUGR + PE—pregnant woman with both intrauterine growth restriction and preeclampsia. The data were interpreted with repeated measures analysis of variance (one way ANOVA) using a post hoc test for multiple comparisons (Dunn’s or Tukey’s tests). The results are expressed as mean ± standard deviation (SD), derived from 2 independent experiments, and were calculated as a percent of control HS (panel A and B) and control HP (panel C and D). Asterisks represent a significant difference when compared with the representative control cells: *p < 0.05, ** *p* < 0.01, *** *p* < 0.001, **** *p* < 0.0001.

The exposure of HUVECs to medium supplemented with human serum taken from healthy pregnant women, pregnant women with IUGR, and IUGR with PE for 24 hours reveals a significant decrease in ECs’ viability in the groups from pregnancies with complications (Figure 1A,C). Serum taken from IUGR and IUGR with PE reduced cell viability by 16% and 17%, respectively, when compared to the cells exposed to serum taken from healthy pregnancies (Figure 1C). Conversely, after the acute exposition (24 h), we did not observe any change in cell proliferation (Figure 2A,C). Prolonged exposition (10 days) caused a significant decrease in cell viability (Figure 1B,D) and proliferation (Figure 2B,D). The effect of reduced cell viability was more pronounced in the group of cells exposed to medium supplemented with sera from IUGR with PE than from IUGR alone either after 1 and 10 days (Figure 1B,D). Sera taken from healthy pregnant women did not change ECs viability and proliferation both in acute and chronic exposition (Figure 1 and Figure 2).

### 3.3. Reactive Oxygen Species (ROS) Generation

**Figure 3:**
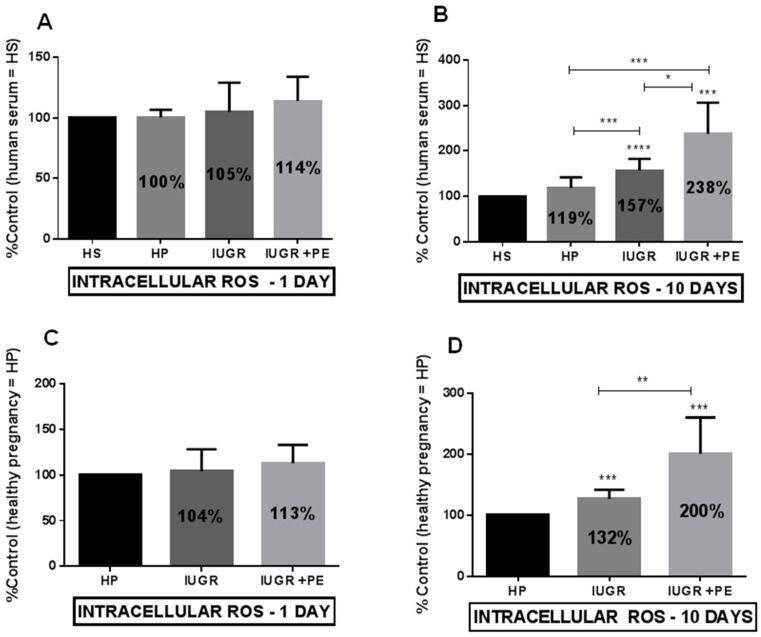
Intracellular reactive oxygen species (ROS) generation by endothelial cell.(**A**) Endothelial cell ROS generation after one day of exposition to medium supplemented with tested sera, expressed as a percent of control which was the human serum taken from non-pregnant women with matched age(HS); (**B**) Endothelial cell ROS generation after ten days of exposition to medium supplemented with tested sera, expressed as a percent of control HS (**C**) Endothelial cell ROS generation after one day of exposition to medium supplemented with tested sera, expressed as a percent of control which was the human serum taken from healthy pregnant women (HP); (**D**) Endothelial cell ROS generation after ten days of exposition to medium supplemented with tested sera, expressed as a percent of control HP. Abbreviations: IUGR—pregnant women with intrauterine growth restriction; IUGR + PE—pregnant woman with both intrauterine growth restriction and preeclampsia. The data were interpreted with repeated measures analysis of variance (one way ANOVA) using a post hoc test for multiple comparisons (Dunn’s or Tukey’s tests). The results are expressed as mean ± standard deviation (SD), derived from 2 independent experiments, and were calculated as a percent of control HS (panel A and B) and control HP (panel C and D). Asterisks represent a significant difference when compared with the representative control cells: *p < 0.05, ** *p* < 0.01, *** *p* < 0.001, **** *p* < 0.0001.

Any of the tested groups generate endothelial ROS production during acute exposition (Figure 3A,C). The process was much more pronounced during the time of exposition and revealed the highest ROS production in the IUGR and PE group (Figure 3B,D). ECs exposed to the medium supplemented with serum taken from healthy pregnant women did not stimulate ROS production during acute and chronic exposition (Figure 3A,B).

### 3.4. Light Microscopy

**Figure 4:**
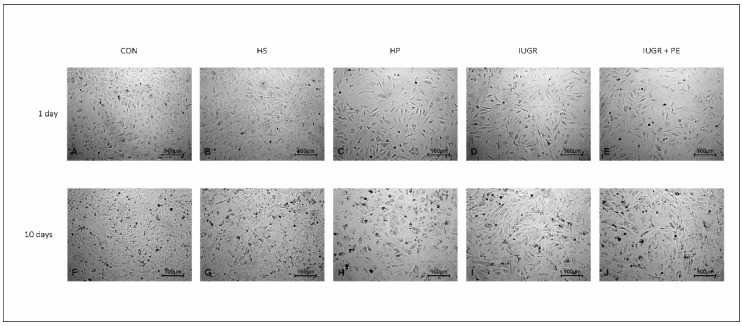
Light microphotographs of human umbilical vein endothelial cell (HUVEC) line EA.hy926 after one and ten days of exposition to medium supplemented with 10% *v/v* human pooled serum taken from: healthy non pregnant women with matched age (HS), healthy pregnant women (HP), pregnant women with intrauterine growth restriction (IUGR), pregnant woman with both intrauterine growth restriction and preeclampsia (IUGR + PE), and to standard medium supplemented with 10% *v/v* fetal bovine serum (CON). Magnification 100×.

We did not observe any morphological changes in ECs after one day of the exposition, using light microscopy. In all tested groups, ECs maintained the typical cobblestone appearance (Figure 4A–E). The first symptoms of irregular shape in IUGR with PE and IUGR alone were observed on the sixth day of the exposition. Chronic exposition revealed morphological changes and decreased cell density in all groups exposed to medium supplemented with human serum (HS, HP, IUGR, IUGR + PE, Figure 4G–J), except the cells exposed to the standard medium (Con) which preserved their typical cobblestone appearance (Figure 4F).

### 3.5. Electron Microscopy

**Figure 5:**
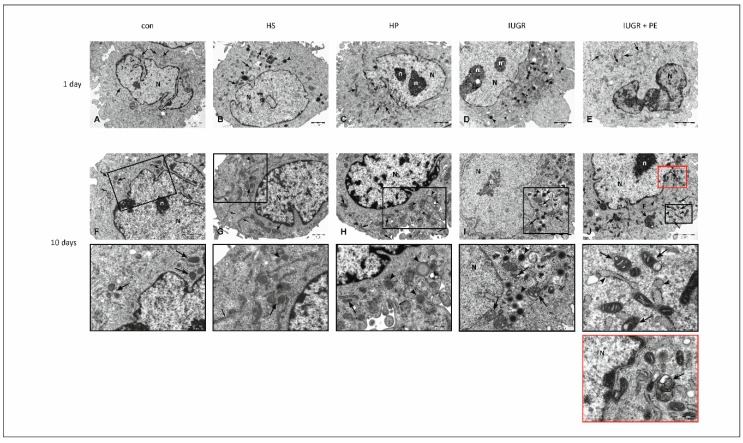
Electron micrographs of EA.hy926 cell line after one (panels **A**–**E**) and 10 (panels **F**–**J**) days of exposition to medium supplemented with: 10% *v/v* human pooled serum taken from: healthy non pregnant women with matched age (HS), healthy pregnant women (HP), pregnant women with intrauterine growth restriction (IUGR), pregnant woman with both intrauterine growth restriction and preeclampsia (IUGR + PE), and to standard medium supplemented with 10% *v/v* fetal bovine serum (Con). After one day of exposure to the modified medium (panels A–E), no significant differences between study groups in the cell ultrastructure were observed. A regularly round-shaped cytoplasm contains numerous mitochondria (arrows), and vesicles with electron-dense material (arrowheads). The majority of cellular organells is localized to the one pole of the nucleus, which is irregular in shape with heterochromatin located at the periphery. Ten days of exposition to modified medium did not reflect the significant changes in the ultrastructural composition of HUVECs derived from Con, HS and HP groups (panels F–H). An irregular in shape nucleus with heterochromatic rim, numerous mitochondria (arrows) with normal ultrastructural features, well developed rough endoplasmatic reticulum (RER, small arrows) were observed. In the cytoplasm of HUVECs from HS (Figure 5G) and HP (Figure 5H) groups, individual lipid droplets were observed (arrow heads). Insets demonstrates magnified regions of the cytoplasm of HUVECs derived from Con, HS and HP groups with typical ultrastructural features indicated with the same signs as on the output figures. Ultrastructural changes in HUVECs derived from IUGR group (Figure 5I) refer mainly to mitochondria—enlarged intermembrane spaces and shape alterations are visible (arrows). In the cytoplasm, numerous lipid droplets (arrowheads) can be observed. The inset presents the magnified region of cytoplasm with modified mitochondria. Figure 5J displays changes in the ultrastructure of HUVECs derived from the IUGR + PE group. The cytoplasm is abundant in organells, which are localized in the close vincinity of the nucleus as well as at the peripheral regions of the cell. Ultrastructural features of mitochondrial swelling can be observed—the mitochondrial matrix is condensed and contains swollen, disrupted cristae (arrows). RER cisterns with few ribosomes possess a dilated lumen containing material of intermediate electron density (arrowheads). In the close neighborhood to the nucleus accumulation of filaments is visible (F). Note autophagic vacuoles with an amorphous electron-dense sub-cellular material are dispersed throughout the cytoplasm (double-headed arrow). Black inset—the enlarged region with swollen mitochondria and RER, red inset—the magnified area of cytoplasm showing autophagic vacuole. N—nucleus, n—nucleolus.

To determine ultrastructural changes in HUVECs’ morphology, transmission electron microscope studies were performed. After one day of exposure to modified medium no significant differences between study groups in the cell ultrastructure were observed (Figure 5A–E). The 10-day period of exposure to the modified medium doesn’t reflect the significant changes in the ultrastructural composition of HUVECs derived from Con, HS and HP groups (Figure 5F–H), while in HUVECs derived from IUGR (Figure 5I) and IUGR with PE (Figure 5J) groups, abnormalities in cellular ultrastructure were observed. They were generally concerned with the (i) mitochondrial structure, (ii) accumulation of lipid droplets in the cytoplasm, and, additionally, in the IUGR with PE group, to (iii) presence of autophagic vacuoles. The precise description of the endothelium is below, Figure 5.

## 4. Discussion

Our study confirmed that patients with IUGR and IUGR with PE are characterized by the impaired function of vascular endothelial cells. During the chronic exposition of ECs in vitro, exposed to the medium supplemented with pooled serum taken from women with complicated pregnancies, we documented the intensification of oxidative stress and reduced cell viability. Additionally, we have observed reduced ECs proliferation which could be partially attributable to decrease pro-angiogenic VEGF found in the serum from complicated pregnancies. The pathological alteration in the placenta stimulates the release of many antiangiogenic factors but VEGF blockade seemed to be crucial for ECs’ proliferation and migration, which may have an adverse effect on the cardiovascular system. Soluble fms-like tyrosine kinase (sFlt)-1 prevents VEGF interaction with receptors located on vascular ECs blocking its stimulation, leading to maternal endothelial dysfunction in pregnancies complicated with IUGR and PE [4,5], but these observations are still obscure [8,9].

The diversity of morphological changes in the ECs was confirmed in the observation of ultrastructure of these cells in an electron microscope. In all tested groups, among cells with typical morphology, distinct ultrastructural changes were observed, especially pronounced in IUGR and IUGR with PE. Observed abnormalities were generally concerned with the accumulation of (i) lipid droplets in the cell cytoplasm, (ii) changes in mitochondrial structure, and additionally in the IUGR with PE group, to (iii) presence of autophagic vacuoles. Among observed ultrastructural changes, abnormalities in mitochondrial structure and the presence of autophagosomes can serve as potent factors leading to cell death. Cell death can be defined as an irreversible loss of plasma membrane integrity. Simplifying, historically three basic forms of cell death have been distinguished in mammalian cells by morphological criteria: necrosis, apoptosis and autophagy [16]. The permeability of mitochondria membranes determines whether cells will succumb to or survive the injury. From the morphologic point of view, the cell exhibits a series of changes that inform us about its stage. Under the light microscope we observed: (i) mild cytoplasmic swelling, (ii) dilatation of organelles, (iii) loss of ribosomes and RER (rough endoplasmic reticulum), (iv) blebbing from the plasma membrane of cytoplasmatic fragments that included cytosol but not the large organelles (mitochondria, endoplasmic reticulum; ER). In the last phase of necrosis, several events were displayed: (i) cytoplasm loses details, (ii) mitochondrial swelling, (iii) visible matrix densities and vacuoles, (iv) deposits of calcium phosphates, and (v) chromatin condensation, fragmentation or dissolution [16,17]. In the groups with complicated pregnancies mitochondrial and ER dysfunctions were predominant. Mitochondria vary considerably in size, structure, and the quantity depending on cell type. They generate most of the cell’s energy (ATP) during oxidative phosphorylation, and are also involved in other tasks, such cellular signaling, differentiation, proliferation, cell aging, apoptosis and death [18]. ATP levels are an essential determinant of whether cells die by apoptosis or necrosis. It is crucial for cell metabolism to keep the integrity of cells and provide cell proliferation, regulation of cell volume, solute concentration (Na, K, Ca), and cellular architecture [19]. Endothelial chronic exposition to tested serum reduced its viability proliferation and increased production of ROS. The fundamental mechanism of these abnormalities could be explained by mitochondrial dysfunction. The relationship between cellular proliferation and mitochondria has also been documented. Cells require an ATP in order to synthesize bioactive compounds such as lipids, proteins, and nucleotides for regular cell proliferation [20]. The variation in ATP levels at different stages of the cell cycle supports the hypothesis that mitochondria play an essential role in cell cycle regulation [21] and determinant of whether cells die by apoptosis or necrosis [18].

Malperfusion of the placenta is a potent inducer of oxidative stress which has been linked to the complication of pregnancy including PE and IUGR [1]. Oxidative stress is defined as a condition in which the generation of reactive oxygen radicals overwhelms a cell’s capacity to detoxify them leading to damage to biological molecules including proteins, lipids, and DNA and consequently leading to ageing and cell death [22]. The principal source of ROS under normal condition is the mitochondria where, during oxidative phosphorylation, a small percentage of electrons may prematurely reduce oxygen, forming ROS such as superoxide [18]. Mitochondria-generated ROS play an essential role in the release of pro-apoptotic proteins (e.g., cytochrome c), which can trigger caspase activation and apoptosis but mitochondrial antioxidant enzymes protect from apoptosis. Hence, there is accumulating evidence supporting a direct link between mitochondria, oxidative stress and cell death [18,23]. ROS are also produced as by-products of detoxification of xenobiotics, and during inflammation [1]. They are not only harmful by-products but are important as a signalling intermediate, regulating the activity of redox-sensitive transcription factors (NF-kB, HIF-1, AP-1) to maintain metabolic homeostasis [24]. ROS can stimulate the secretion of pro-inflammatory cytokines and are responsible for the senescent-associated secretory phenotype (SASP) [1,23]. The assay we use to evaluate ROS production in the endothelium is not really specific for mitochondrial ROS but also detects ROS from other sources, and indicates that tested sera have pro-oxidative potential. Normal pregnancy represents a state of oxidative stress caused by increased maternal metabolism and metabolic activity of the placenta. Although the generation of ROS is increased during pregnancy [25], the placental production of ROS is usually counterbalanced by antioxidants [1]. ROS generation is increased during hypoxia and ischemia reperfusion [26]. In defective placentation, and decreased utero-placental vascularisation, the periods of placental ischemia/reperfusion and hypoxia favor oxidative stress [27]. Holland et al. demonstrated that placentas in women who reached the term and those who delivered a pre-term response to preeclampsia in a different way have the potential to release adaptive mechanisms. The adaptive changes concerned mitochondria-related ROS production and, availability of antioxidants, which are triggered by oxygen deprivation (hypoxia/reoxygenation) [28]. In PE the level of antioxidants may be too low to contribute to the increased ROS production [29]. The oxidative stress is not only generated by mitochondria but also by ER. There is a close physical and functional association between mitochondria and the ER. Calcium signalling and transfer of lipids integrate the two organelles [30,31]. Mitochondrial biogenesis strictly depends on the import of ER-synthesized lipids [32]. The ER is also a source of enzymes (heme oxygenases; HO) that inhibit oxidative stress, inflammation, and apoptosis [33] while in mitochondria superoxide dismutase (SOD) is responsible for superoxide anion scavenging. These two organelles contribute to increased production of ROS during malperfusion in preeclamptic placentas [1,4]. Expression of antioxidant enzymes (SOD, HO, glutathione peroxidase, catalase) is decreased in PE. Moreover, the total antioxidant capacity is lower in the serum of PE than in healthy pregnant women [29,34].

Mitochondrial swelling is a hallmark of mitochondrial dysfunction and is one of the most important indicators of the opening of the mitochondrial permeability transition pore. It is a cell-specific ultrastructural change visualized by electron microscopy, which allows the detection of temporal dynamics of mitochondrial swelling in living specimens [35]. Dysfunctional mitochondria observed in groups with complicated pregnancies are the primary source of ROS in endothelium but as reported by Wen et al. ROS itself could be the reason to form swollen and ruptured mitochondria in ECs [36]. Mitochondria participate in numerous biochemical pathways (oxidative phosphorylation, cellular signaling, differentiation, proliferation, aging, apoptosis, cell death) and, therefore, are essential for the viability of cells. In our study, we observed a decrease in ECs viability in the groups with IUGR and IUGR with PE, during short and chronic exposure. Our viability test (MTT) is based on the activity of dehydrogenases which are present in various cellular organelles such as cytoplasm, mitochondria, ER, and others [14] and appears to be a very sensitive assay, showing subtle changes in cell viability after 24 hours of exposition. These changes were not yet visible in the microscopic observations (light and electron) after this time.

In electron microscopy, we frequently observed lipid droplets (LDs), characteristic for metabolic dysfunction. Its presence could be partly explained by the disability of ER and mitochondria, especially in IUGR and PE groups. LDs store energy as triacylglycerols and sterol esters and are initiated in the ER. They dynamically interact with ER, mitochondria, lysosomes, and peroxisomes. They grow in size and number due to increased triacylglycerols synthesis when cells enter the stationary phase. Utilization of LDs is driven by the hydrolysis of triacylglycerols during growth resumption [37]. The existence of undefined factor X, released in the abnormal placenta, generates not only oxidative stress but also interferes with lipid metabolism. In PE lipid levels are increased even more than in healthy pregnancy [38]. Incubation of ECs with PE sera increased their triglyceride content [39]. One more observation was to detect the presence of autophagic vacuoles only in endothelium exposed to medium supplemented with serum from IUGR with the PE group. Zhang et al. documented that the autophagy process is involved in the degradation of oxy-LDL in HUVECs by the autophagy-lysosomal pathway [40]. Autophagy is a highly regulated process that may be included in the turnover of long-lived proteins and organelles and may help cells survive in an unfavorable environment to protect against apoptosis, but it can also lead to cell destruction [41,42]. Parts of the cytoplasm and intracellular organelles are sequestered within characteristic double-membrane autophagic vacuoles (known as autophagosomes) and are ultimately delivered to lysosomes for bulk degradation (for more details see [43]). Autophagy is critical in lipid metabolism and could have important implications for human disease with lipid over-accumulation [44].

## 5. Conclusions

Maternal constitutional factors (genetic, immunological, pre-existing vascular diseases) are involved in defective placentation. Hypoperfusion of the placenta releases many factors seem to be involved in vascular endothelial dysfunction and clinical symptoms of PE, and often in IUGR. Endothelial dysfunction presented in complicated pregnancies as IUGR and PE has its bases in cell ultrastructural changes, mainly related to mitochondria but also to the accumulation of lipid droplets and autophagic vacuoles formation. These lesions could be responsible for the abnormal endothelial secretory phenotype, angiogenic properties, and its capability to produce ROS. Deterioration of endothelial functions leads to clinical symptoms characteristic for cardiovascular diseases as hypertension, proteinuria, activation of pro-coagulant activity, inflammation, and incorrect redistribution of fluids. Our results can help to understand the nature of the disorders studied in pregnant women and contribute to their more effective treatment.

## Figures and Tables

**Table 1:** Clinical and biochemical characteristic of study subjects. The data were interpreted with repeated measures analysis of variance (one-way ANOVA or Kruskal–Wallis) using a post hoc test for multiple comparisons (Tukey’s test or Dunn’s test). Abbreviations: IUGR—pregnancy complicated with intrauterine growth restriction of the fetus, IUGR + PE—pregnancy complicated with intrauterine growth restriction and preeclampsia, SYS—systolic blood pressure, DIA—diastolic blood pressure, hsCRP—high-sensitivity C-reactive protein, VEGF—vascular endothelial growth factor.

Parameters	Normal Pregnancy Control (*n* = 25)	IUGR (*n* = 25)	IUGR + PE (*n* = 24)
Age, years	32 ± 6	29 ± 6	30 ± 6
Number of pregnancy, median (min-max)	2 (1–8)	1 (1–8)	1 (1–5)
Primiparous, *n* (%)	7 (28%)	14 (56%)	13 (34%)
Gestational age, week, median (min-max)	39 (38–41)	36 (28–40)	34 (28–40)
Newborn weight, g	3531 ± 476	2032 ± 720 ****	1734 ± 684 ****
SYS, mmHg	109.6 ± 6.6	112.9 ± 21.7 ††††	132.9 ± 18.6 ****
DIA, mmHg	69.2 ± 5.53	68.6 ± 9.7 ††††	82.7 ± 12.86 **
Urine protein, g/L / 24 h	below 0.03	0,15 ± 0 ††††	3.07 ± 4.31 ****
hsCRP, mg/L	5.3 ± 3.4	6.2 ± 9.0	5.4 ± 4.7
VEGF, pg/mL	139.0 ± 37.5	36.3 ± 20.2 ****	40.9 ± 2.2 ****

Significance difference vs. control: ** *p* < 0.01, **** *p* < 0.0001. Significance difference vs. IUGR + PE: †††† *p* < 0.0001.

## References

[1] Burton G.J., Jauniaux E. (2011). Oxidative stress. Best Pract. Res. Clin. Obstet. Gynaecol..

[2] Roland C.S., Hu J., Ren C.E., Chen H., Li J., Varvoutis M.S., Leaphart L.W., Byck D.B., Zhu X., Jiang S.W. (2016). Morphological changes of placental syncytium and their implications for the pathogenesis of preeclampsia. Cell. Mol. Life Sci..

[3] Bonello S., Zahringer C., Belaiba R.S., Djordjevic T., Hess J., Michiels C., Kietzmann T., Gorlach A. (2007). Reactive oxygen species activate the HIF-1alpha promoter via a functional NFkappaB site. Arterioscler. Thromb. Vasc. Biol..

[4] Burton G.J., Jauniaux E. (2018). Pathophysiology of placental-derived fetal growth restriction. Am. J. Obstet. Gynecol..

[5] Maynard S.E., Min J.Y., Merchan J., Lim K.H., Li J., Mondal S., Libermann T.A., Morgan J.P., Sellke F.W., Stillman I.E. (2003). Excess placental soluble fms-like tyrosine kinase 1 (sFlt1) may contribute to endothelial dysfunction, hypertension, and proteinuria in preeclampsia. J. Clin. Investig..

[6] Jonsdottir L.S., Arngrimsson R., Geirsson R.T., Sigvaldason H., Sigfusson N. (1995). Death rates from ischemic heart disease in women with a history of hypertension in pregnancy. Acta Obstet. Gynecol. Scand..

[7] Venkatesha S., Toporsian M., Lam C., Hanai J., Mammoto T., Kim Y.M., Bdolah Y., Lim K.H., Yuan H.T., Libermann T.A. (2006). Soluble endoglin contributes to the pathogenesis of preeclampsia. Nat. Med..

[8] Yinon Y., Kingdom J.C., Odutayo A., Moineddin R., Drewlo S., Lai V., Cherney D.Z., Hladunewich M.A. (2010). Vascular dysfunction in women with a history of preeclampsia and intrauterine growth restriction: Insights into future vascular risk. Circulation.

[9] Crispi F., Dominguez C., Llurba E., Martin-Gallan P., Cabero L., Gratacos E. (2006). Placental angiogenic growth factors and uterine artery Doppler findings for characterization of different subsets in preeclampsia and in isolated intrauterine growth restriction. Am. J. Obstet. Gynecol..

[10] Roberts J.M., Edep M.E., Goldfien A., Taylor R.N. (1992). Sera from preeclamptic women specifically activate human umbilical vein endothelial cells in vitro: Morphological and biochemical evidence. Am. J. Reprod. Immunol..

[11] Matsubara K., Matsubara Y., Hyodo S., Katayama T., Ito M. (2010). Role of nitric oxide and reactive oxygen species in the pathogenesis of preeclampsia. J. Obstet. Gynaecol. Res..

[12] Wang Y., Gu Y., Zhang Y., Lewis D.F. (2004). Evidence of endothelial dysfunction in preeclampsia: Decreased endothelial nitric oxide synthase expression is associated with increased cell permeability in endothelial cells from preeclampsia. Am. J. Obstet. Gynecol..

[13] Edgell C.J., McDonald C.C., Graham J.B. (1983). Permanent cell line expressing human factor VIII-related antigen established by hybridization. Proc. Natl. Acad. Sci. USA.

[14] Mosmann T. (1983). Rapid colorimetric assay for cellular growth and survival: Application to proliferation and cytotoxicity assays. J. Immunol. Methods.

[15] Witowski J., Korybalska K., Wisniewska J., Breborowicz A., Gahl G.M., Frei U., Passlick-Deetjen J., Jorres A. (2000). Effect of glucose degradation products on human peritoneal mesothelial cell function. J. Am. Soc. Nephrol..

[16] Krysko D.V., Vanden B.T., D’Herde K., Vandenabeele P. (2008). Apoptosis and necrosis: Detection, discrimination and phagocytosis. Methods.

[17] Vanlangenakker N., Vanden B.T., Krysko D.V., Festjens N., Vandenabeele P. (2008). Molecular mechanisms and pathophysiology of necrotic cell death. Curr. Mol. Med..

[18] Ott M., Gogvadze V., Orrenius S., Zhivotovsky B. (2007). Mitochondria, oxidative stress and cell death. Apoptosis.

[19] Pedersen P.L. (1994). ATP synthase. The machine that makes ATP. Curr. Biol..

[20] Antico Arciuch V.G., Elguero M.E., Poderoso J.J., Carreras M.C. (2012). Mitochondrial regulation of cell cycle and proliferation. Antioxid. Redox Signal..

[21] Sweet S., Singh G. (1999). Changes in mitochondrial mass, membrane potential, and cellular adenosine triphosphate content during the cell cycle of human leukemic (HL-60) cells. J. Cell. Physiol..

[22] Genc H., Uzun H., Benian A., Simsek G., Gelisgen R., Madazli R., Guralp O. (2011). Evaluation of oxidative stress markers in first trimester for assessment of preeclampsia risk. Arch. Gynecol. Obstet..

[23] Huang H., Manton K.G. (2004). The role of oxidative damage in mitochondria during aging: A review. Front. Biosci..

[24] Kohen R., Nyska A. (2002). Oxidation of biological systems: Oxidative stress phenomena, antioxidants, redox reactions, and methods for their quantification. Toxicol. Pathol..

[25] Myatt L., Webster R.P. (2009). Vascular biology of preeclampsia. J. Thromb. Haemost..

[26] Guzy R.D., Schumacker P.T. (2006). Oxygen sensing by mitochondria at complex III: The paradox of increased reactive oxygen species during hypoxia. Exp. Physiol..

[27] Sanchez-Aranguren L.C., Prada C.E., Riano-Medina C.E., Lopez M. (2014). Endothelial dysfunction and preeclampsia: Role of oxidative stress. Front. Physiol..

[28] Holland O.J., Cuffe J.S.M., Dekker N.M., Callaway L., Kwan Cheung K.A., Radenkovic F., Perkins A.V. (2018). Placental mitochondrial adaptations in preeclampsia associated with progression to term delivery. Cell Death Dis..

[29] Nakamura M., Sekizawa A., Purwosunu Y., Okazaki S., Farina A., Wibowo N., Shimizu H., Okai T. (2009). Cellular mRNA expressions of anti-oxidant factors in the blood of preeclamptic women. Prenat. Diagn..

[30] Hayashi T., Rizzuto R., Hajnoczky G., Su T.P. (2009). MAM: More than just a housekeeper. Trends Cell Biol..

[31] Huang C.Y., Chiang S.F., Lin T.Y., Chiou S.H., Chow K.C. (2012). HIV-1 Vpr triggers mitochondrial destruction by impairing Mfn2-mediated ER-mitochondria interaction. PLoS ONE.

[32] Klecker T., Westermann B. (2014). Mitochondria are clamped to vacuoles for lipid transport. Dev. Cell.

[33] Dulak J., Loboda A., Jozkowicz A. (2008). Effect of heme oxygenase-1 on vascular function and disease. Curr. Opin. Lipidol..

[34] Turpin C.A., Sakyi S.A., Owiredu W.K., Ephraim R.K., Anto E.O. (2015). Association between adverse pregnancy outcome and imbalance in angiogenic regulators and oxidative stress biomarkers in gestational hypertension and preeclampsia. BMC Pregnancy Childbirth.

[35] Gerencser A.A., Doczi J., Torocsik B., Bossy-Wetzel E., dam-Vizi V. (2008). Mitochondrial swelling measurement in situ by optimized spatial filtering: Astrocyte-neuron differences. Biophys. J..

[36] Wen Y.D., Wang H., Kho S.H., Rinkiko S., Sheng X., Shen H.M., Zhu Y.Z. (2013). Hydrogen sulfide protects HUVECs against hydrogen peroxide induced mitochondrial dysfunction and oxidative stress. PLoS ONE.

[37] Barbosa A.D., Siniossoglou S. (2017). Function of lipid droplet-organelle interactions in lipid homeostasis. Biochim. Biophys. Acta Mol. Cell Res..

[38] Hubel C.A., Roberts J.M., Taylor R.N., Musci T.J., Rogers G.M., McLaughlin M.K. (1989). Lipid peroxidation in pregnancy: New perspectives on preeclampsia. Am. J. Obstet. Gynecol..

[39] Lorentzen B., Endresen M.J., Hovig T., Haug E., Henriksen T. (1991). Sera from preeclamptic women increase the content of triglycerides and reduce the release of prostacyclin in cultured endothelial cells. Thromb. Res..

[40] Zhang Y., Sun J., Yu X., Shi L., Du W., Hu L., Liu C., Cao Y. (2016). SIRT1 regulates accumulation of oxidized LDL in HUVEC via the autophagy-lysosomal pathway. Prostaglandins Other Lipid Mediat..

[41] Dong G., Yang S., Cao X., Yu N., Yu J., Qu X. (2017). Low shear stressinduced autophagy alleviates cell apoptosis in HUVECs. Mol. Med. Rep..

[42] Wang S., Sun X., Jiang L., Liu X., Chen M., Yao X., Sun Q., Yang G. (2016). 6-Gingerol induces autophagy to protect HUVECs survival from apoptosis. Chem. Biol. Interact..

[43] Mizushima N. (2007). Autophagy: Process and function. Genes Dev..

[44] Singh R., Kaushik S., Wang Y., Xiang Y., Novak I., Komatsu M., Tanaka K., Cuervo A.M., Czaja M.J. (2009). Autophagy regulates lipid metabolism. Nature.

